# DeepImmuno: deep learning-empowered prediction and generation of immunogenic peptides for T-cell immunity

**DOI:** 10.1093/bib/bbab160

**Published:** 2021-05-03

**Authors:** Guangyuan Li, Balaji Iyer, V B Surya Prasath, Yizhao Ni, Nathan Salomonis

**Affiliations:** University of Cincinnati, 3333 Burnet Ave, MLC7024, Cincinnati, OH 45267, USA; University of Cincinnati, USA; Biomedical Informatics, Cincinnati Children’s Hospital Medical Center, USA; Cincinnati Children’s Hospital Medical Center, USA; Cincinnati Children’s Hospital Medical Center, USA

**Keywords:** immunogenicity, deep learning, convolutional neural network, generative adversarial network, COVID-19, neoantigen

## Abstract

Cytolytic T-cells play an essential role in the adaptive immune system by seeking out, binding and killing cells that present foreign antigens on their surface. An improved understanding of T-cell immunity will greatly aid in the development of new cancer immunotherapies and vaccines for life-threatening pathogens. Central to the design of such targeted therapies are computational methods to predict non-native peptides to elicit a T-cell response, however, we currently lack accurate immunogenicity inference methods. Another challenge is the ability to accurately simulate immunogenic peptides for specific human leukocyte antigen alleles, for both synthetic biological applications, and to augment real training datasets. Here, we propose a beta-binomial distribution approach to derive peptide immunogenic potential from sequence alone. We conducted systematic benchmarking of five traditional machine learning (ElasticNet, K-nearest neighbors, support vector machine, Random Forest and AdaBoost) and three deep learning models (convolutional neural network (CNN), Residual Net and graph neural network) using three independent prior validated immunogenic peptide collections (dengue virus, cancer neoantigen and SARS-CoV-2). We chose the CNN as the best prediction model, based on its adaptivity for small and large datasets and performance relative to existing methods. In addition to outperforming two highly used immunogenicity prediction algorithms, DeepImmuno-CNN correctly predicts which residues are most important for T-cell antigen recognition and predicts novel impacts of SARS-CoV-2 variants. Our independent generative adversarial network (GAN) approach, DeepImmuno-GAN, was further able to accurately simulate immunogenic peptides with physicochemical properties and immunogenicity predictions similar to that of real antigens. We provide DeepImmuno-CNN as source code and an easy-to-use web interface.

## INTRODUCTION

Immunotherapy has emerged as a promising strategy to combat cancer by ‘reprogramming’ a patient’s own immune system. Effective targeted immunotherapies require accurately predicting which cancer-specific neo-peptides are most likely to elicit an immune response. Similar strategies are currently being designed to target antigens commonly produced by serious pathogens, such as the SARS-CoV-2 (COVID-19) virus [1]. Human leukocyte antigens (HLAs) are a polymorphic class of proteins on the cell surface of all human nucleated cells that present foreign antigens to T-cell receptors (TCRs). The process of antigen recognition is the cornerstone of the adaptive immune system. HLA proteins are encoded by the major histocompatibility complex (MHC) genes in humans. Predicting the immunogenicity of MHC-I bound peptides is crucial for understanding the molecular rules governing T cell-directed adaptive immunity and creating precision cancer- or pathogen-targeted vaccines. Cellular antigen recognition is governed by a series of carefully orchestrated molecular interactions between cell surface-presented antigens and T cells of the immune system. MHC-I proteins are responsible for the presentation of short peptides on the cell surface and mediating interactions with TCRs on CD8+ T cells. A T cell-specific immune response will be triggered if a peptide is capable of binding with a cognate MHC molecule, and the resultant peptide–MHC complex can further interact with selected TCR sequences. Peptides that meet these criteria are referred to as immunogenic peptides. The exposure of ‘foreign’ signals triggers immunoreceptor tyrosine-based activation motifs on the T cell to be phosphorated and activates an immune response [2]. The process ultimately results in targeted cell death of the antigen-expressing cell by the CD8+ T cell. Hence, the identification of immunogenic epitopes that can trigger T-cell responses is central to developing new cancer immunotherapies and vaccines. Because thousands of potential disease-associated antigens can be presented in innate or foreign cells [3], it is necessary to prioritize which candidates are most likely to induce a T-cell response prior to experimental validation.

The accurate prediction of immunogenicity requires knowledge of (i) which peptides are likely to bind MHC and (ii) which peptide–MHC pairs will activate an immune response. A plethora of HLA-peptide binding prediction tools has been developed to predict which peptides will bind to specific cognate HLA alleles (donor-specific). However, MHC-binding prediction alone is insufficient to infer immunogenicity as such tools do not model which peptides will trigger a T-cell response [2, 4–6]. To fill the gap and complement the current MHC-binding prediction tools, *in silico* methods have been developed to predict antigen immunogenicity. POPI [7] was developed as the first automated computational immunogenicity prediction tool. POPI used a selected subset of physicochemical features identified by a bi-objective algorithm for support vector machine (SVM)-based classification. An updated version POPISK [8] further considers MHC binding properties to improve its prediction ability. PAAQD [9] was later developed to consider amino acid pairwise contact potential and quantum topological molecular similarity for feature selection. Subsequently, a machine learning-based immunogenicity predictor NeoPepsee [10] was developed that integrated 14 independent features to infer peptide immunogenicity. These initial methods paved the way for more advanced algorithms; however, the applicability of such methods has historically been challenging due to small training datasets and limited consideration of HLA alleles. A significant advance in the field came with the introduction of the immune epitope database (IEDB) and associated immunogenicity prediction tools [5]. This invaluable resource continues to systematically characterize the biochemical properties of over 30 000 MHC-I-bound immunogenic peptides. IEDB further includes a suite of algorithms to predict binding affinity and immunogenicity, including a position-weighted calculated schema by considering Kullback–Leibler divergence and amino acid preference (default method). More recently, algorithms with improved reported accuracy have been described, including a Random Forest-based approach called INeo-Epp [11], which uses a customized immunogenic score, and the recurrent neural network-based deep learning approach DeepHLApan [12]. While promising, a potential limitation of these approaches is that the prediction of immunogenic epitopes is treated as a binary classification problem using predefined hard cutoffs, in which each peptide–MHC pair will be considered immunogenic or non-immunogenic, even though the immunogenicity of a certain peptide–MHC will vary substantially depending on the subject’s immune profile and TCR repertoire [2]. Further, while DeepHLApan [12] applies a well-rationalized deep learning approach, its encoding of the amino acid sequence does not incorporate physicochemical or other amino acid parameters (one-hot encoding). As a result, the outputs from these methods might not fully reflect the ability of the peptide–MHC to trigger a T-cell response.

A secondary, but important challenge in the field of immunogenicity prediction, is to learn the rules that govern which peptides are immunogenic and why. Understanding these rules could be used to develop improved prediction models or produce large synthetic datasets for training more accurate predictive models or conversely identify peptides with a low likelihood of inducing an immune response in engineered genomes. Deep generative models [13] are a newly emerging area in artificial intelligence that can be applied to diverse research problems. In effect, such methods allow for the creation of accurate synthetic models from limited existing training data. Such methods take random noise to create new datasets that reflects the original training data and that contains unique informative features. Generative adversarial networks (GANs) are widely used in computer vision [14] and synthetic biology [15] to generate new images or sequences of interest (i.e. antimicrobial peptides) but have not previously used to produce synthetic models of immunogenic peptides.

To overcome the aforementioned limitations, we propose a new convolutional neural network (CNN) [16] approach called DeepImmuno-CNN. Rather than predict MHC–peptide interactions, this tool predicts immunogenicity of MHC–peptide pairs. During the training, a beta-binomial probabilistic model is fitted to the training dataset to derive a continuous immunogenic score. Unlike other immunogenicity prediction methods, this score differentially weights each peptide–MHC complex in the model based on the strength of available experimental immunogenicity evidence in our training dataset (high confidence or low confidence). Each amino acid sequence is additionally encoded using a reduced principal component analysis (PCA) feature space of 566 well-curated amino acid physicochemical features from the AAindex1 database [17] to overcome sparsity issues related to one-hot encoding [18]. Diverse machine learning and deep learning approaches exist, which have potential strengths and weaknesses for this problem (e.g. performance, accuracy, flexibility to dataset size). To ensure the rigor of this approach, we performed a systematic comparison of five traditional machine learning algorithms [ElasticNet, K-nearest neighbors (KNN), SVM, Random Forest and AdaBoost] and three deep learning models [CNN, graph neural network (GNN), Residual Net (ResNet)]. This benchmarking further supports the use of a CNN for this problem. In addition, an evaluation of different encoding schemas confirms that our AAindex1 PCA encoding strategy provides excellent performance relative to alternative methods. When benchmarked against two state-of-the-art workflows for immunogenicity prediction (DeepHLApan and IEDB), DeepImmuno-CNN was able to significantly increase both precision and recall for different HLA genotypes using diverse real-world testing datasets (dengue, cancer neoantigen and SARS-CoV-2). To further explore the dependent peptide features for immunogenicity prediction, we developed a GAN model [15, 19], which mimics the salient features of validated immunogenic peptides. These data support the hypothesis that immunogenic peptides are learnable as a possible future source for high-quality synthetic training data.

## METHODS

### Datasets

For initial training and validation, we analyzed >9000 tested immunogenicity molecular assays from the Immune Epitope Database, IEDB database (13 August 2020). We restricted this dataset to peptides with metadata that matched the following keywords: (i) linear epitope, (ii) T-cell assay, (iii) MHC class I, (iv) human and (v) any disease. To only consider informative predictions, we applied a rigorous data cleaning strategy. First, data instances without explicit 4-digit MHC alleles were discarded. Second, all redundant peptide–MHC allele instances were discarded (the same peptide with different HLA alleles were considered different instances). Third, all negative peptides, without explicit experimental information (number of subjects tested, number of subjects responded) or with less than four tested subjects were removed (likely not informative at a human population level). Fourth, peptides of lengths 9 and 10 were retained for training. 9-mer and 10-mer peptides cover 97.5% of all data instances and are also the dominant length for MHCI-bound peptides [20]. Finally, we separated out 408 dengue virus positive instances from Weiskopf *et al*. [21] for the purpose of initial validation of different prediction methods. Specifically, 8971 data instances were retained in the final dataset, among which 4059 were positive reactive instances and the remaining 4912 were negative. We used 10-fold cross-validation for initial benchmarking to avoid overfitting, in which we split the datasets into 10 rotating subsets—9 for training and 1 for validation in each run. At the end of cross-validation, the scores for each evaluation metric were averaged over the 10 testing subsets as the model’s performance. We selected two independent test datasets for further evaluation: (i) 608 experimentally tested tumor-specific neoantigens from the Tumor Neoantigen Selection Alliance (TESLA) [22] and (ii) 100 SARS-CoV-2 peptides [1, 22] tested for their immunogenicity in convalescent and unexposed subjects, respectively. The detailed descriptions for each validation dataset and the experiments used for generating them are shown in Supplementary Table S3 available online at https://academic.oup.com/bib.

Additional algorithm, evaluation and website development details are provided in Supplemental Methods available online at https://academic.oup.com/bib.

## RESULTS

DeepImmuno-CNN was developed with the primary objective of improving immunogenicity predictions, separate from MHC binding, for relevant disease antigens identified from diverse upstream approaches. To this end, we set out to systematically evaluate existing as well as potential machine and deep learning strategies. This benchmarking was performed on multiple recently described high-quality experimentally validated immunogenic peptides, after carefully excluding low-confidence experimental results (Methods).

### Evaluation criteria

We used different evaluation metrics depending on the characteristics of each testing dataset. For the tumor neoantigen test dataset, we considered a restricted dataset of the (i) top 20 or (ii) top 50 immunogenic peptides predicted by each algorithm or (iii) overall sensitivity. The top 20 or 50 immunogenic peptides were purposely selected as these are the same number of peptides considered in prior reports [22]. For the sensitivity analysis, a threshold of 0.5 was used for DeepImmuno-CNN and DeepHLApan and a threshold of 0 for the IEDB default classification algorithm, which has a distinct scoring range. Since an absolute threshold is not used for DeepImmuno-CNN, which outputs a score based on the trained binomial distribution, this threshold was only used for comparative benchmarking purposes. It is worth noting that we do not consider specificity in the validated neoantigen dataset because each peptide has only been tested in a single cancer patient and hence it is highly likely that a certain peptide can be immunogenic in a larger population with more diverse TCR repertoires.

For antigens from a recent COVID-19 study, we considered recall and precision as the primary criteria due to a much higher number of observed negative versus positive immunogenic antigens (imbalanced). For initial evaluation, we used 10-fold cross-validation to assess the effectiveness of DeepImmuno-CNN. In each iteration, the area under the receiver operating characteristic curve (auROC) and area under the precision-recall curve (auPR) were computed to compare performance at different selected cutoffs. auPR is more informative than auROC in an imbalanced scenario due to the incorrect interpretation of specificity [23]. For the five evaluated machine learning algorithms, we tuned the major hyperparameters based on 10-fold cross-validation with root mean square error (RMSE) as the evaluation criterion.

### Comparison of immunogenicity prediction models

**Figure 1:**
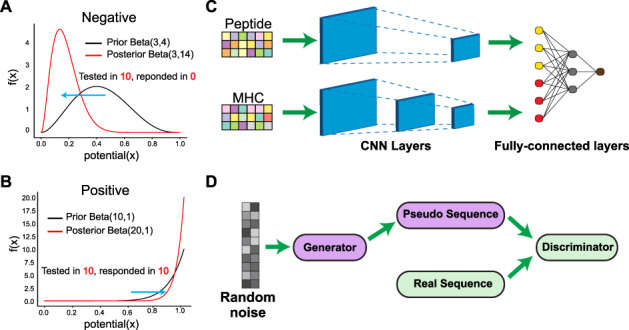
The DeepImmuno model. (**A**, **B**) In DeepImmuno, to assess the probability that a given antigen is immunogenic, variable peptide immunogenic potential is computed by sampling from a posterior beta distribution of well-defined true-positive and true-negative immunogenic antigens to produce a continuous immunogenic score. The posterior distribution is derived using a subset of T-cell immunogenic assay results from the Immune Epitope Database (binomial) and a prior beta distribution of either (**A**) negative or (**B**) positive assay results. (**C**) The DeepImmuno-CNN architecture is shown to predict immunogenicity for each peptide–MHC complex. In this model, each peptide–MHC pair is subjected to two consecutive convolutional layers, followed by two fully connected dense layers to output a predictive value for each pair. (**D**) The DeepImmuno-GAN architecture is depicted for simulating immunogenic peptide sequences using only random sequences as an input. The GAN model is composed of a generator and a discriminator. This learning generator produces pseudo-sequences to artificially convince the discriminator that the immunogenic sequences are real, while the discriminator uses real peptide sequences along with generated pseudo-sequences to distinguish the difference.

To account for the variable immunogenic potential for each evaluated peptide, we fitted a beta-binomial probabilistic model in the training dataset to derive a continuous immunogenic score (Figure 1A and B and Methods). For instance, the peptide RPIDDPFGL for the HLA allele HLA-B^*^0702 was tested in 40 subjects and triggered a T-cell response in all 40 subjects, whereas the peptide KTWGQYWQV in conjunction with HLA-A^*^0201 elicited a T-cell response in only 1 out of 6 subjects, even though both are ‘immunogenic’. While this score does not reflect the genuine biochemical immunogenicity strength, it does reflect the statistical confidence in our model based on the relative experimental evidence in our training dataset. Additionally, it does not consider MHC binding, as the training dataset is biased towards already predicted MHC-bound peptides. Given that significant biases exist in the training data, in terms of how many times a peptide was tested in different contexts or with different assays, increased modeling confidence will exist for peptides most similar to those with high-quality data during training.

To select the best predictive model, we constructed five traditional machine learning regressors (ElasticNet, KNN, SVM, Random Forest and AdaBoost) and critical hyperparameters were tuned via cross-validation (Supplemental Methods available online at https://academic.oup.com/bib). In addition, we explored the potential of three deep learning models (CNN, ResNet and GNN). We systematically gauged their performance in three testing datasets (dengue virus [21], tumor neoantigens [22] and SARS-CoV-2 [1]) (Supplementary Table S2 available online at https://academic.oup.com/bib). The Random Forest-based regressor had a slightly better RMSE in the nested 10-fold validation than other models, and AdaBoost regression performed the best in the dengue virus dataset with average accuracy = 0.91. However, the CNN model achieved superior performance in the neoantigen dataset, where it predicted 2.9 and 5.9 immunogenic epitopes on average in its top 20 and top 50 predictions, respectively. All the models achieved similar results on the SARS-CoV-2 dataset with an average recall of around 0.72 in convalescent patients and 0.81 in the unexposed groups. Given that it is able to mimic the interaction between peptide and MHC, we designed a graph CNN model; however, it suffered from ‘shortcut learning’ [24] such that all the predictive values are around 0.5, in order to achieve a lower loss during the training stage. This can be attributed to the fact that the explicit weight assignments in the graph may not entirely reflect the real peptide–MHC interactions, which in turn can lead to ambiguous results. To explore whether increasing the complexity of the neural network architecture can boost performance, we constructed a ResNet model, with 12 layers and skip connections. As ResNet did not increase the performance and had inferior results in eight out of nine evaluation criteria across three testing datasets, we surmise that a more complex model is not required. Considering its performance overall and in human disease datasets, adaptability to training datasets of variable size and the complexity of the model, we chose CNN as the optimal prediction model for further analysis, which we call hereafter DeepImmuno-CNN. As a final consideration, we attempted to validate our proposed amino acid encoding strategy, which considers both indices derived from amino acid physicochemical properties (AAindex) and HLA allotype information (paratopes). While the use of these algorithms did not result in significant performance boosts with neural network-based approaches over alternative strategies, our selected encoding methods did not decrease performance and did offer a performance boost for specific machine learning methods (Random Forest) for specific test datasets, suggesting its benefits may be situation-dependent (Supplementary Table S2 and Supplementary Figure S3 available online at https://academic.oup.com/bib).

**Figure 2:**
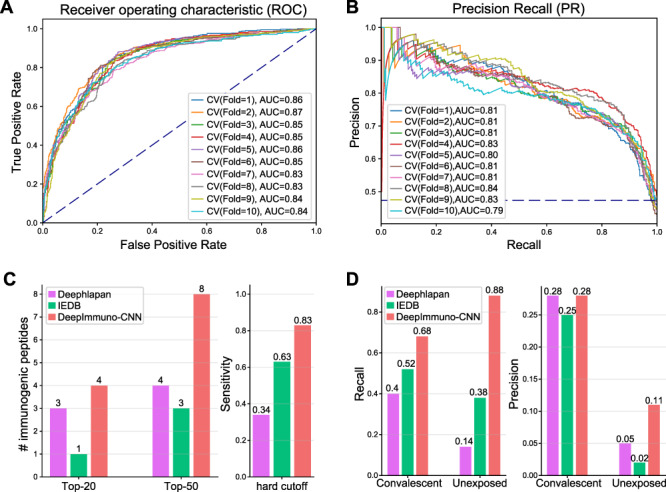
DeepImmuno-CNN produces stable predictions and outperforms existing methods. The (**A**) ROC curve and (**B**) precision-recall curve of only DeepImmuno-CNN on 10-fold validation of the IEDB training dataset. Here, 0.5 is used as the threshold to discriminate between positive and negative instances. (**C**) Comparison of immunogenicity predictions from an experimentally validated tumor neoantigen dataset (608 tested), with the number of true-positive predictions overlapping with each algorithm’s top 20 or top 50 predictions (left), or the sensitivity of each algorithm using a 0.5 scoring threshold (right). (**D**) In the COVID-19 study, recall (left) and precision (right) of each algorithm in convalescent COVID-19 patients and the unexposed individuals using a 0.5 scoring threshold.

To validate the effectiveness of the DeepImmuno-CNN model, we conducted 10-fold cross-validation in the IEDB dataset, on its own (Figure 2A and B). As noted above, an absolute threshold is required (0.5) for certain tested algorithms to predict if a peptide is immunogenic or non-immunogenic. We found DeepImmuno-CNN to be highly stable with a high average auROC (0.85) and auPR (0.81) for each fold. We next compared the performance of this CNN model relative to other prior described immunogenicity prediction methods, specifically DeepHLApan and IEDB (default algorithm), as these methods are well validated and have easy-to-use interfaces. When evaluated in the tumor neoantigen dataset, DeepImmuno-CNN found an impressive 29 out of 35 (83%) immunogenic neoantigens, relative to IEDB which found 63%, and DeepHLApan which only found (34%) out of a total of 608 antigens experimentally tested (Figure 2C). For the same neoantigen dataset, DeepImmuno-CNN predicts 4 in the top 20 and 8 in the top 50 neoantigens, while IEDB performed relatively poorly (1 in the top 20 and 4 in the top 50), with DeepHLApan producing intermediate results (Figure 2C). To compare these results to random, we shuffled the labels during training and found that all nine evaluated metrics performed lower with these prediction algorithms (Supplementary Figure S4 available online at https://academic.oup.com/bib).

We further evaluated DeepImmuno-CNN using a recently published COVID-19 study, where immunogenic peptides were validated from two groups of subjects. Convalescent patients have already been infected by SARS-CoV-2 and are in the process of recovering, while unexposed patients have not been infected. In both convalescent and unexposed groups, DeepImmuno-CNN achieved the highest sensitivity (68% in convalescent, 88% in unexposed) compared to IEDB (52% in convalescent, 38% in unexposed) and DeepHLApan (40% in convalescent, 14% in unexposed) (Figure 2D). DeepImmuno-CNN also achieved the highest precision (0.28 in convalescent, 0.11 in unexposed), with an overall low precision due partially to the fact that COVID-19 patients are a highly selective group and their unique immune profile might not be representative of the whole population. To determine if increasing the training time improves overall performance, we further tested epochs beyond the original default upper limit (64, 100, 150). While all models produce consistent results in the datasets tested (Supplementary Figure S4 available online at https://academic.oup.com/bib), increasing the training time reduced the false-positive and false-negative rates when running DeepImmuno-CNN on the entire IEDB training dataset (Supplementary Figure S4 available online at https://academic.oup.com/bib). Hence, we use an epoch of 150 as the revised DeepImmuno-CNN default to minimize underfitting observed at lower epochs.

We next looked for potential immunodominant regions in the SARS-CoV-2 proteome, which can be exploited for T-cell vaccine development. Our results suggest that both 9-mers and 10-mers do not predict immunodominant regions in general (Supplementary Figure S5 available online at https://academic.oup.com/bib). To understand how evolving mutations in SARS-CoV-2 may enhance viral transmission, we compared DeepImmuno-CNN predictions in these variants [25–27]. Our analysis finds that while CD8+ T-cell immunogenicity is position- and HLA-dependent for D614G and E484K, the N501Y mutation shows a widespread increase in immunogenicity (Supplementary Figure S6 available online at https://academic.oup.com/bib). These findings present new possible avenues for T cell-based vaccine approaches for emerging SARS-CoV-2 variants.

### DeepImmuno-CNN reveals salient positions interacting with the TCR

**Figure 3:**
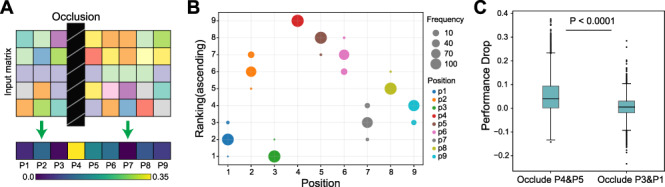
Identification of salient immunogenic features of peptide–TCR interactions. (**A**) Schematic overview of the occlusion sensitivity technique to determine the relative contribution of each antigen residue for the DeepImmuno-CNN model predictive score. (**B**) Ascending importance rank of each position, with the position with the largest performance drop receiving the highest ranking across 100 simulations. Dot size corresponds to the frequencies of each position assigned the denoted rank, with different colors indicating different amino acid positions. (**C**) Performance decrease for the occlusion of P4 + P5 with occlusion of P3 + P1. One-sided Mann–Whitney *U* test *P*-value (*P* = 7.94e−209).

To understand the molecular underpinnings of DeepImmuno-CNN’s predictions, we examined the dependency of this model on each residue position using occlusion sensitivity. The largest decrease in performance corresponds to the most important position across the peptide as shown in a saliency heatmap (Figure 3A). The saliency of positions can be attributed to both peptide–HLA interaction and peptide–TCR interaction. We simulated this process 100 times and an ascending ranking was performed each time to highlight the most salient positions, as shown in (Figure 3B). This analysis reveals that amino acid positions P4 (residue 4), P5 and P6 are consistently the most dependent positions, followed by P2, P8 and P9. Occlusion of the first and second most dependent positions (P4 and P5) compared to the least (P3 and P1) resulted in a significant performance drop of every single positive instance (One-sided Mann–Whitney *U* test, *P*-value = 7.9e−209; Figure 3C). These results support prior structural prediction studies, which show that P4–6 interacts with the TCR with the greatest frequency [28, 29], whereas P2 and P9 serve as anchor points for binding of the peptide–MHC complex [5] and mirrors other computational predictions [8].

To assess the rules governing T-cell immunogenicity for different HLA alleles, we next evaluated MHC allele dependence on specific amino acid preferences. To perform this analysis, we collected all immunogenic peptides bound with each allele and derived a motif matrix based on the inferred position importance weight in the model (Supplemental Methods and Supplementary Figure S7 available online at https://academic.oup.com/bib). For example, when examining the allele HLA-A^*^0201, we find leucine is the most abundant amino acid in position 2 from the model, which is consistent with prior structural evidence [30]. Similarly, Hu *et al*. [31] found that positions 2 and 9 were predicted to act as anchor points for interactions with this specific HLA allele. Our motif matrix additionally suggests that positions 4 and 5 interact with the TCR on the other side. We conducted the same analysis on three other HLA alleles (HLA-A^*^2402, HLA-B^*^0702 and HLA-B^*^0801). These alleles were chosen because the number of associated immunogenic peptides bound to these three alleles is greater than 150, suggesting that the immunogenic motif matrix for these alleles is stable. As expected, position 4 also shows a stronger pattern across these three alleles, compared to other positions, supporting a similar model of HLA–TCR interactions.

### DeepImmuno-GAN accurately mimics immunogenic peptide sequences

To better understand the molecular interactions and biochemical properties of T-cell immunogenicity, we attempted to generate *de novo* immunogenic peptides using a GAN-based approach. Successful creation of such peptides would indicate that immunogenic sequence motifs are learnable, potentially paving the way for direct synthesis and optimization of peptides for diverse applications (e.g. enhanced immunogenicity, non-reactive peptides) [32].

**Figure 4:**
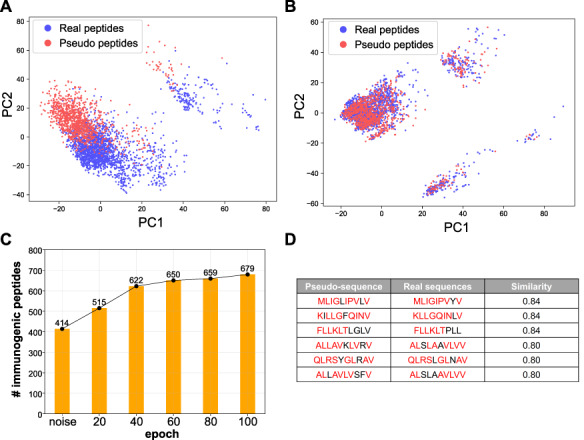
DeepImmuno-GAN is able to learn and produce synthetic immunogenic pseudo-sequences. PCA of the distribution of real sequences (blue dots) and random generative sequences (red dots) (**A**) prior to training and (**B**) after training (100 epochs). The degree of common embedding is considered an indicator of prediction similarity. (**C**) The number of DeepImmuno-CNN predicted immunogenic peptides, produced from noise in different GAN training epochs. (**D**) Example generative pseudo-sequences and their most similar counterparts in experimentally observed HLA-A^*^0201 immunogenic peptides.

As a proof-of-concept, we collected all immunogenic peptides known to bind to HLA-A^*^0201 (the most abundant allele in the training database) for training the deep GAN model. We trained a Wasserstein GAN model for 100 epochs (Supplemental Methods available online at https://academic.oup.com/bib) and extracted the generative pseudo-sequences from every 20 epochs. We utilized the same encoding schema we used in the prediction model to perform dimension reduction using PCA and visualized the distribution of generative and real immunogenic sequences (Figure 4A and B; Supplementary Figure S8A available online at https://academic.oup.com/bib). When viewed as a PCA projection, we find that random peptide sequences significantly deviate from the experimentally validated immunogenic peptide sequences, prior to GAN model training. However, after GAN model training, the generative pseudo-sequence maps to a common coordinate embedding within the PCA projection to that of real immunogenic peptide sequences. These data suggest that the GAN model is able to extract high-level features from real instances and teach the generator to output similar immunogenic peptides built from the random sequence as a starting input. The same distribution shifts were observed with tSNE dimensionality reduction (Supplementary Figure S8B available online at https://academic.oup.com/bib).

To further assess the immunogenicity of these generative sequences, we submitted all generated sequences at different epoch points to our DeepImmuno-CNN model. In the beginning, the 1024 random sequences were found to only contain 40% of the immunogenic sequence (predictive score >0.5). As training progresses, the fraction of immunogenic peptides gradually increases to 67%, which translates to 265 more immunogenic peptides generated during training (Figure 4C). We compared each generative pseudo-sequence to their most similar real counterparts (Figure 4D). The similarity was defined as the total longest contiguous matching subsequence between the real and pseudo-sequence, with 87% (891/1024) of all pseudo-sequences having >60% similarity to their matched real immunogenic peptides (Supplemental Methods and Supplementary Figure S9 available online at https://academic.oup.com/bib) [33]. Hence, immunogenic peptides can be learned and produced when sufficient training data exist. To ensure that the GAN-based model is not simply overfitting its own discriminator, we increased the training time to 100 to 1000 epochs. This analysis finds that the GAN model does not learn to simply reproduce the original training data but rather gets closer to the training probability distributions (Supplementary Figure S9 available online at https://academic.oup.com/bib). Finally, application of our occlusion analysis to GAN-generated immunogenic data (epoch 1000) finds that, similar to DeepImmuno-CNN predictions, P4 and P2 are the most salient positions for HLA-A^*^0201 immunogenic epitopes (Supplementary Figure S9 available online at https://academic.oup.com/bib).

### Online web interface

**Figure 5:**
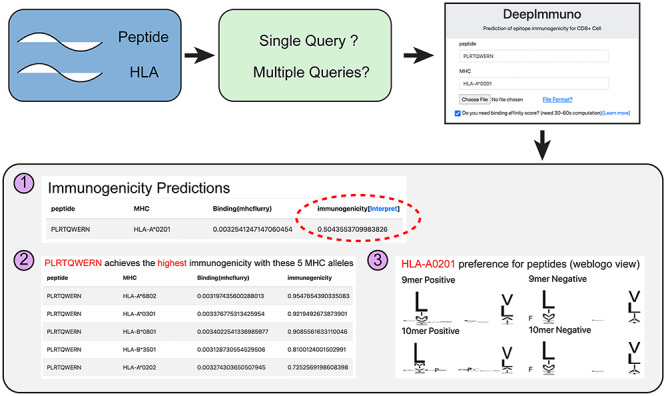
The DeepImmuno web interface. An easy-to-use web interface for querying peptide and HLA sequence pairs. The three primary outputs of the interface are (1) immunogenicity score and MHC-binding potential (optional) for queried peptide-HLA combination, (2) the top five HLA combinations that will yield the highest immunogenicity score for each queried peptide, and the (3) preferential motif of the queried HLA allele.

We developed an easy-to-use web interface allowing users to quickly query peptide sequences to predict immunogenicity potential for given HLA alleles. Additionally, this service allows a user to query for which HLA allele would yield the highest immunogenicity and hence which patients might benefit most from an immunogenic therapy. Additionally, users can determine which peptide a specific HLA allele will prefer or disfavor. This analysis will return the immunogenicity score, top five combinations for different HLA alleles and a weblogo view of all immunogenic and non-immunogenic peptides associated with a certain HLA allele (Figure 5). Moreover, the DeepImmuno web portal allows users to perform multiple queries by specifying an input file with peptide sequence information. Finally, to determine which peptides will both bind MHC and are immunogenic, we incorporated the open-sourced HLA-binding prediction tool MHCflurry [34] to provide coincident prediction of MHC binding.

## DISCUSSION

The accurate identification of potential immunogenic epitopes remains a significant challenge for understanding the molecular mechanisms underlying host immune response and designing effective targeted therapies. Given the fact that millions of possible peptides can be generated from human protein-coding genes [3], experimentally validating all possibilities is simply not yet feasible. Effective computational models can largely accelerate this process by providing a pre-screening platform to find high-confidence immunogenic epitopes or to eliminate low confidence predictions. While current HLA-binding prediction tools have greatly aided in this effort, peptide–MHC binding is insufficient to predict which peptides will produce downstream immune responses [35]. Hence, integrated approaches are required to predict which peptides will bind MHC and activate an immune response. Machine learning and deep learning algorithms have been shown to provide increased performance in a wide spectrum of bioinformatics applications [36, 37]. However, comprehensive benchmarking and the selection of an optimal encoding strategy are required to develop improved models that can be applied to diverse testing datasets.

In this manuscript, we developed a beta-binomial model to generate more accurate immunogenicity potential by considering the overall quality of each experimentally tested antigen in the training dataset. Using these optimized training datasets, we systematically benchmarked well-established machine learning and deep learning, and encoding strategies on independent immunogenic disease datasets, to understand the different situations in which these methods boost, decrease or do not impact overall classification performance. From this extensive comparison analysis, we found that a CNN model in combination with a psychometric-aware encoding strategy balanced performance across diverse test datasets while staying robust for different training dataset sizes. Indeed, we found that increasingly complex deep learning models, such as ResNet, could result in overfitting in this specific application. Our DeepImmuno-CNN model was able to significantly outperform two existing highly used immunogenicity prediction workflows, in terms of overall sensitivity and the top-ranked hits, when applied to diverse real-world immunogenic antigen datasets, including cancer and COVID-19 infection. From a neoantigen pre-screening perspective, DeepImmuno-CNN is most likely to increase the sensitivity for detection of valid neoantigens, such as tumor-specific mutations or splicing neojunctions, from large-scale genomics assays to be tested in downstream assays. Using this optimized model, we were able to effectively identify the most salient residues for interactions between peptide–MHC and TCR, which were recapitulated and added to prior knowledge. Moreover, we developed a GAN modeling approach to accurately generate immunogenic peptides from random noise and demonstrated that the biochemical interactions were learnable given sufficient training data.

Despite these advances described herein, several challenges remain in the field of immunogenicity prediction. While our model significantly improves upon existing approaches in terms of sensitivity, precision and recall, it is noteworthy that all existing approaches remain challenged by lower than preferred specificity to select immunogenic antigens with high confidence. We further note that DeepImmuno-CNN yields a 0.12 false-positive rate and 0.05 false-negative rate in the training dataset and an artificially higher immunogenicity score in certain cases (i.e. KTWGQYWQV, with more sparse experimental evidence, receives a score of 0.8). This limitation could be due to the fact that few disease antigens have been thoroughly tested for their ability to mount a T-cell response in large patient cohorts to ensure reproducibility and HLA allele coverage. However, it is noteworthy that an indispensable component of epitope recognition is the sequence of the TCR, which has not been taken into consideration due to the fact that there exist few matched TCR sequencing data for forming a sufficiently powered training set [38, 39]. Although new high throughput methods for single-cell TCR sequencing have been developed, such techniques are still infrequently performed in research and clinical settings. The increased use of such techniques is likely to aid in the development of more accurate predictive models. In addition, neoantigen T-cell responses can significantly vary from patient-to-patient, due to a variety of factors including immune cell repertoire differences that impact the diversity of activated T-cell clones [40–42]. Hence, validated immunogenic epitopes may be ineffective in a subset of patients. Limited experimental data will account for some false-positive predictions, which may indeed be immunogenic in a subset of patients. Quantitative data on a patient’s TCR repertoire and associated immune responses are likely to significantly improve existing models. We note that currently DeepImmuno-CNN is not capable of discriminating between HLA-bound and unbound peptides. To enable such predictions in the future, we require more unambiguous experimental training data that consider binding strength, stability, immunogenicity and TCR sequences. Beyond providing a rubric for the design of peptide-related models, we believe our approach can be significantly extended to encode additional variables, such as TCR sequence heterogeneity, and can be generalized to address diverse sequence-predictive analyses, beyond immunogenicity.

**Key Points**

- Systematic benchmarking of peptide immunogenicity prediction demonstrates vastly improved performance with DeepImmuno-CNN.
- Generative adversarial network approach to accurately simulate immunogenic peptides.
- Prior-reported salient peptide positions and motifs can be predicted from this CNN.
- Online interface for automated prediction of peptide immunogenicity with DeepImmuno.

## Supplementary Material

FigureS1_bbab160

FigureS2_bbab160

FigureS3_bbab160

FigureS4_bbab160

FigureS5_bbab160

FigureS6_bbab160

FigureS7_bbab160

FigureS8_bbab160

FigureS9_bbab160

Table_S1_bbab160

Table_S2_bbab160

Table_S3_bbab160

Table_S4_bbab160

Supplemental_Methods-r2_bbab160

## Data Availability

DeepImmuno Python3 code is available at https://github.com/frankligy/DeepImmuno. The DeepImmuno web portal is available from https://deepimmuno.research.cchmc.org. The data in this article are available in GitHub and supplementary materials. The source code for reproducing all figures is provided at https://github.com/frankligy/DeepImmuno/tree/main/reproduce/fig.

## Notes

**Guangyuan Li** is a PhD student at the University of Cincinnati. His research interests include developing computational tools in cancer immunotherapy.

**Balaji Iyer** is a PhD student in electrical engineering and computer science at the University of Cincinnati. His research interests are in machine learning applications in bioinformatics and imaging informatics.

**Surya Prasath** is an assistant professor in Biomedical Informatics, Cincinnati Children’s Hospital Medical Center. His research interests are in machine learning and biomedical imaging informatics.

**Yizhao Ni** is an assistant professor at Cincinnati Children’s Hospital Medical Center. His research interests are in machine learning and natural language processing.

**Nathan Salomonis** is an associate professor at Cincinnati Children’s Hospital Medical Center. His research interests are computational genomics and cancer informatics.
