# Supplementary figures and images for: DeepImmuno: deep learning-empowered prediction and generation of immunogenic peptides for T-cell immunity
Source: Brief Bioinform. 2021 May 3;22(6):bbab160. doi: 10.1093/bib/bbab160 (PMC8135853; doi:10.1093/bib/bbab160)

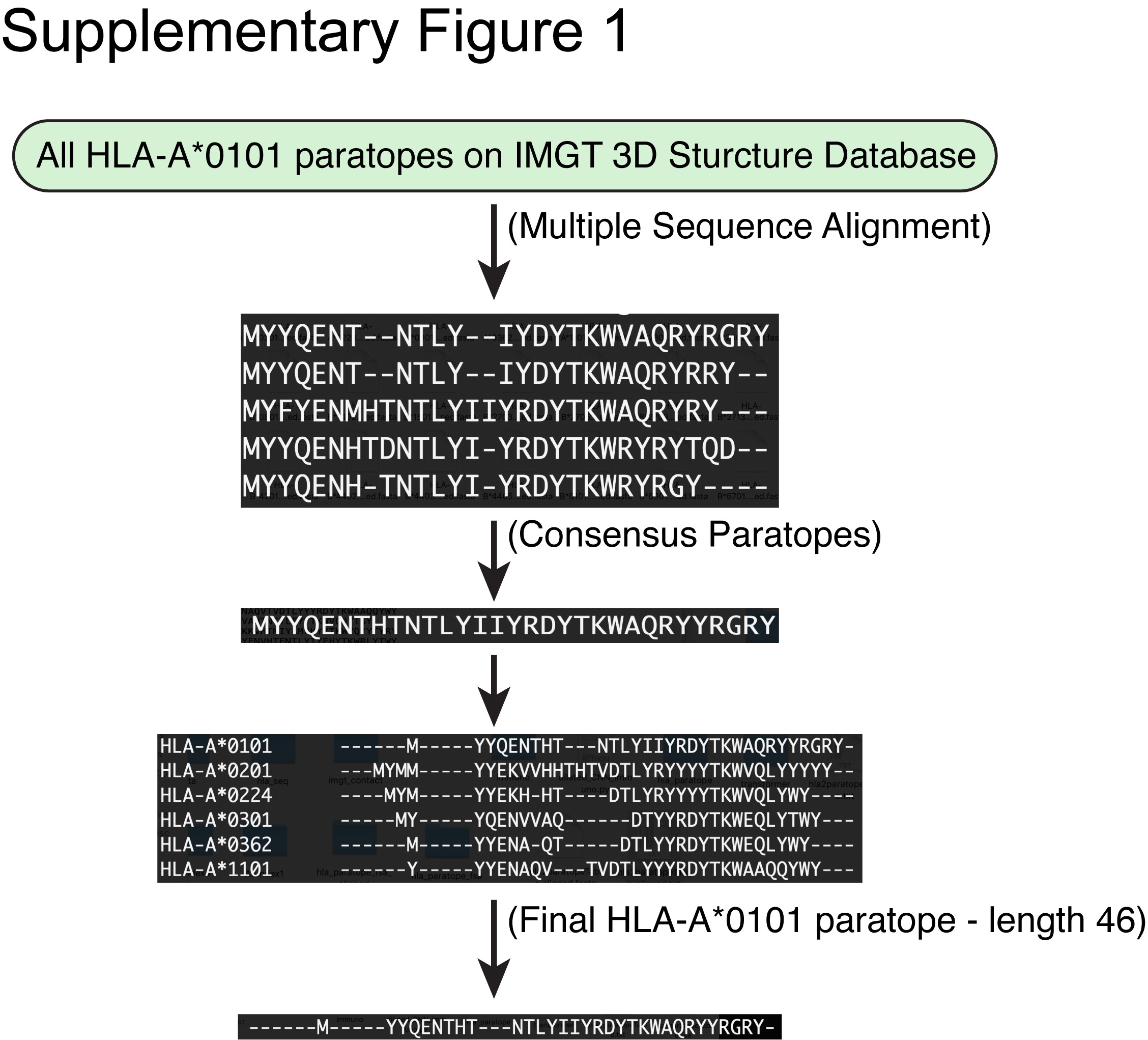

Supplement: FigureS1_bbab160 [file figures1_bbab160.jpeg]

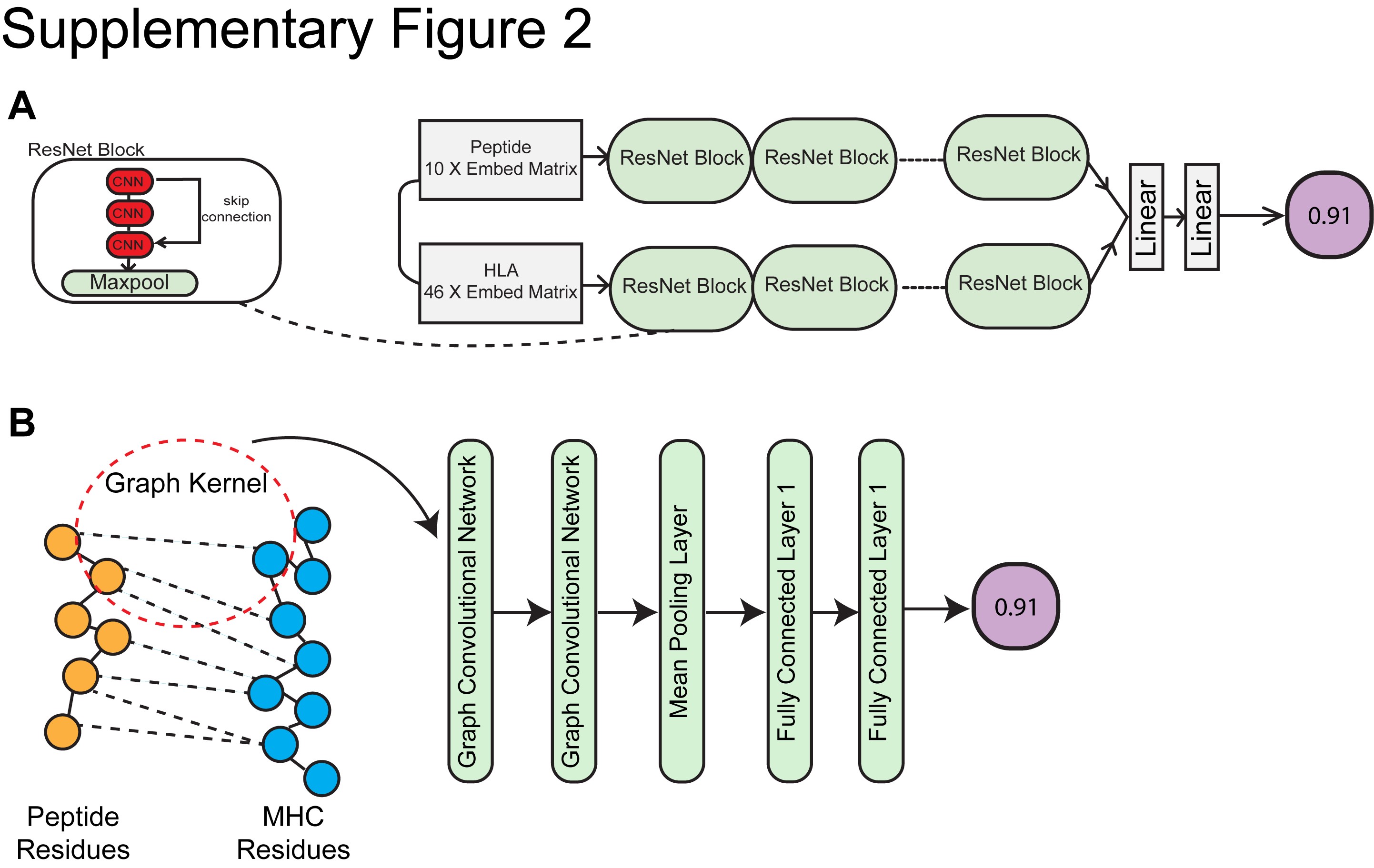

Supplement: FigureS2_bbab160 [file figures2_bbab160.jpeg]

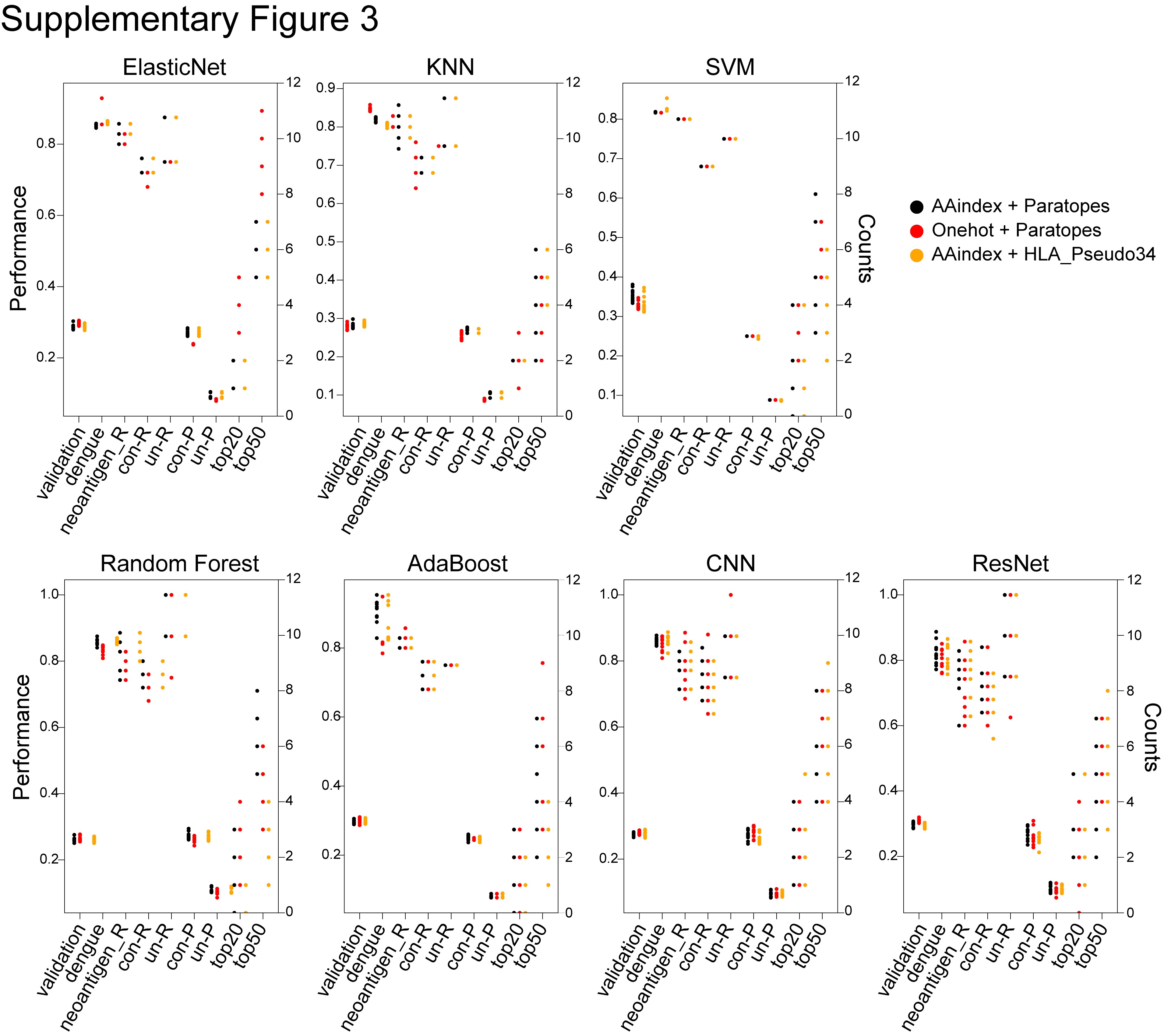

Supplement: FigureS3_bbab160 [file figures3_bbab160.jpeg]

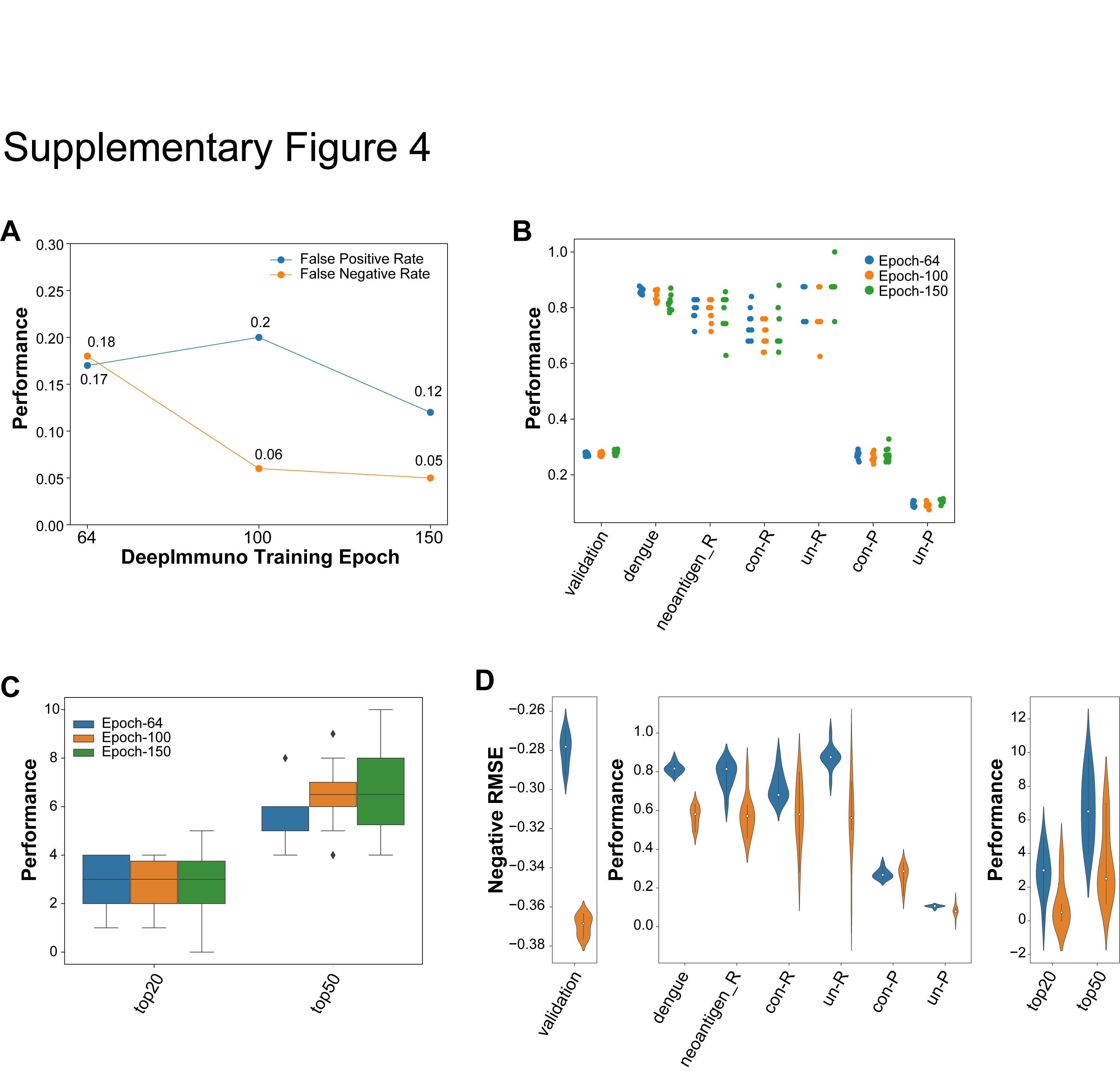

Supplement: FigureS4_bbab160 [file figures4_bbab160.jpeg]

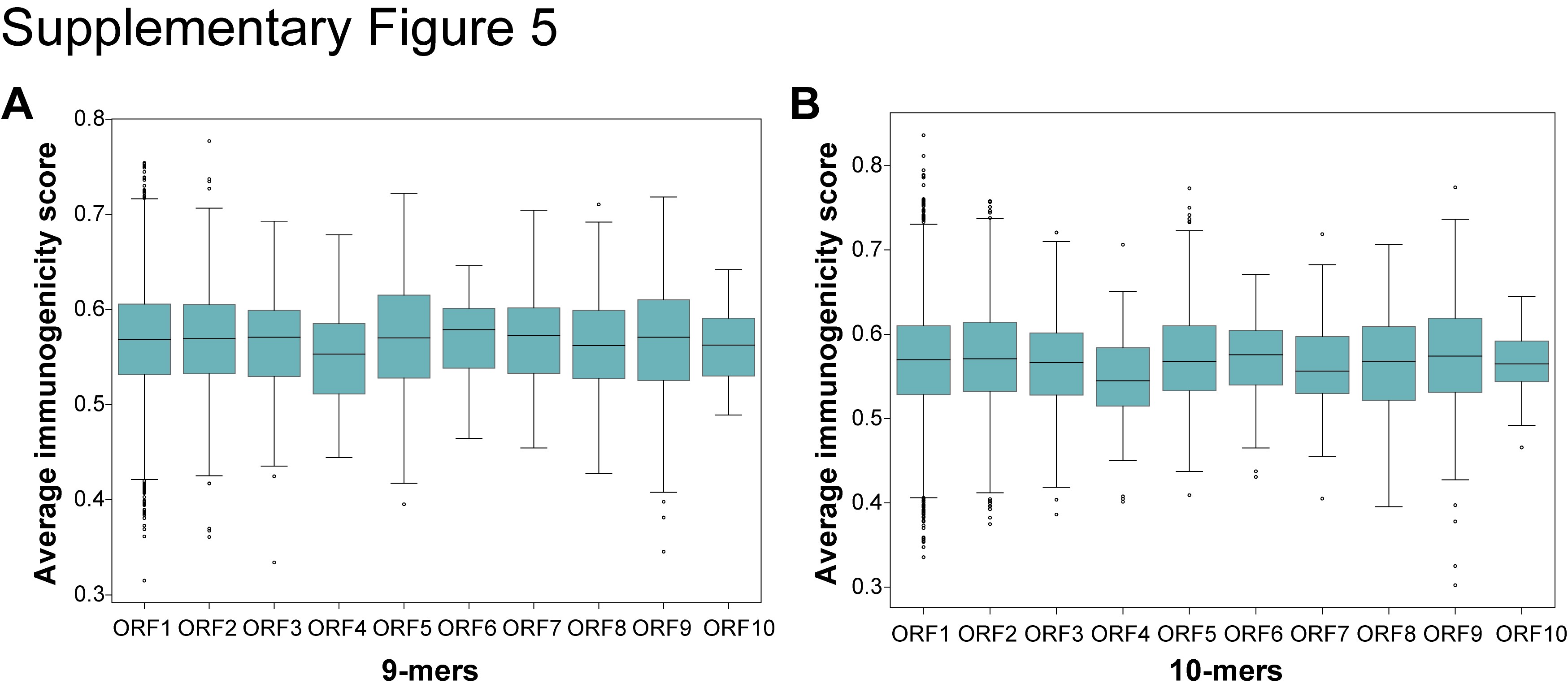

Supplement: FigureS5_bbab160 [file figures5_bbab160.jpeg]

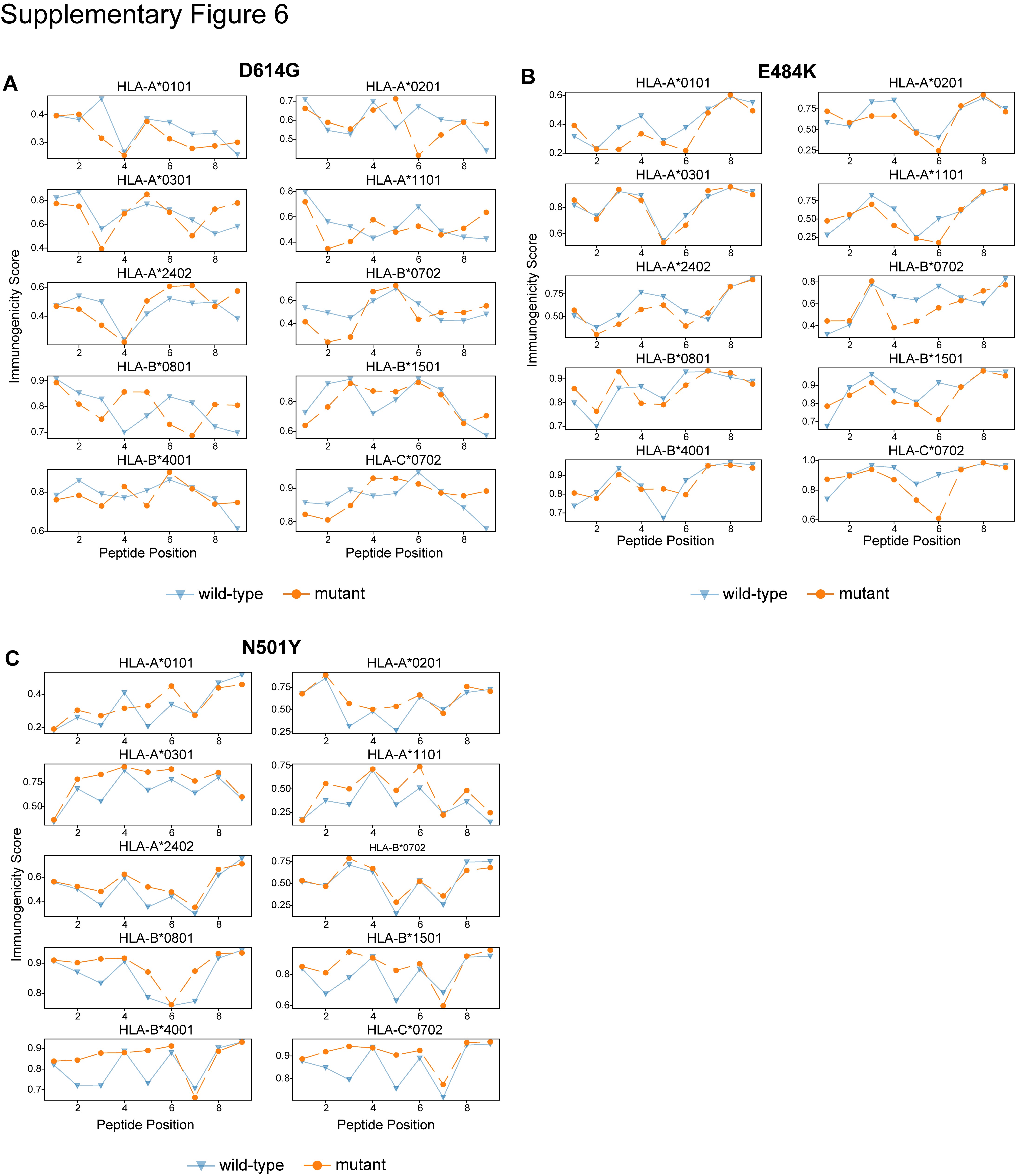

Supplement: FigureS6_bbab160 [file figures6_bbab160.jpeg]

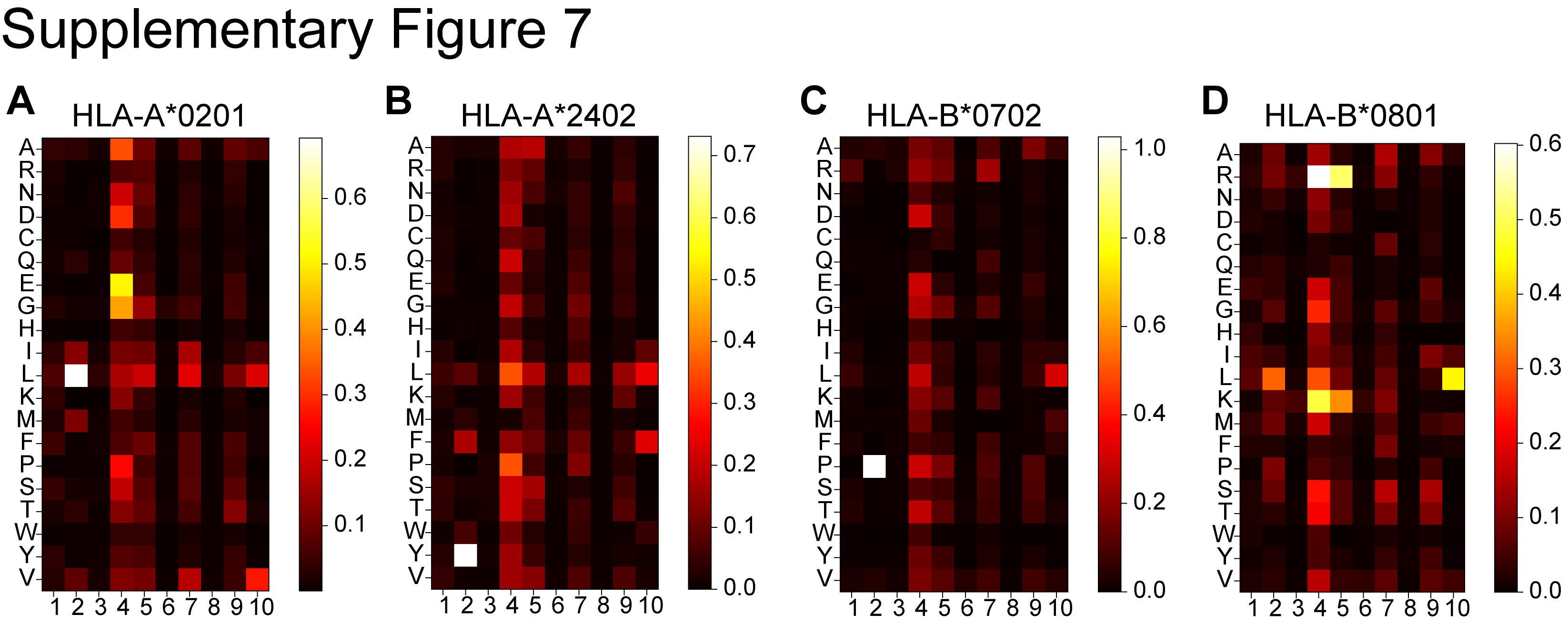

Supplement: FigureS7_bbab160 [file figures7_bbab160.jpeg]

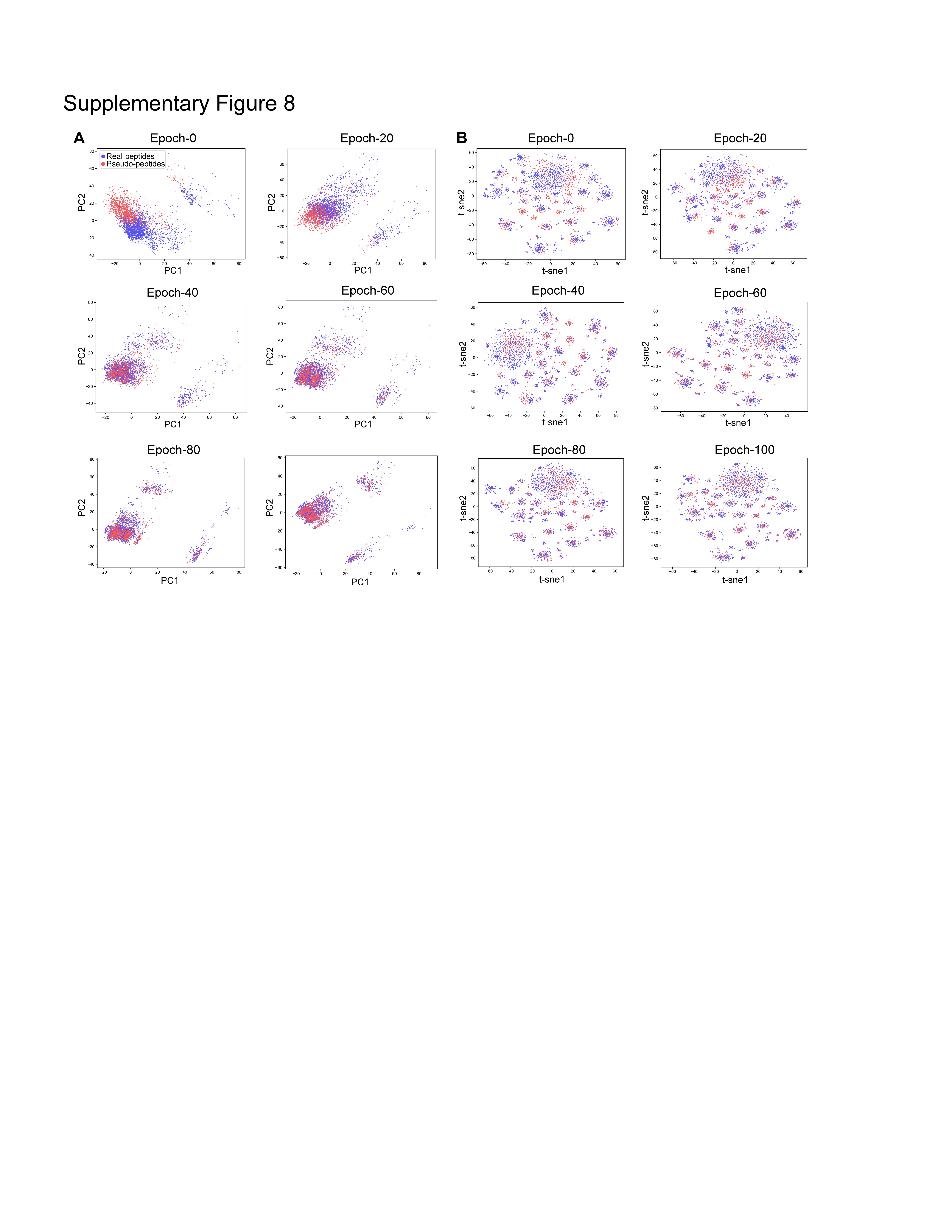

Supplement: FigureS8_bbab160 [file figures8_bbab160.jpeg]

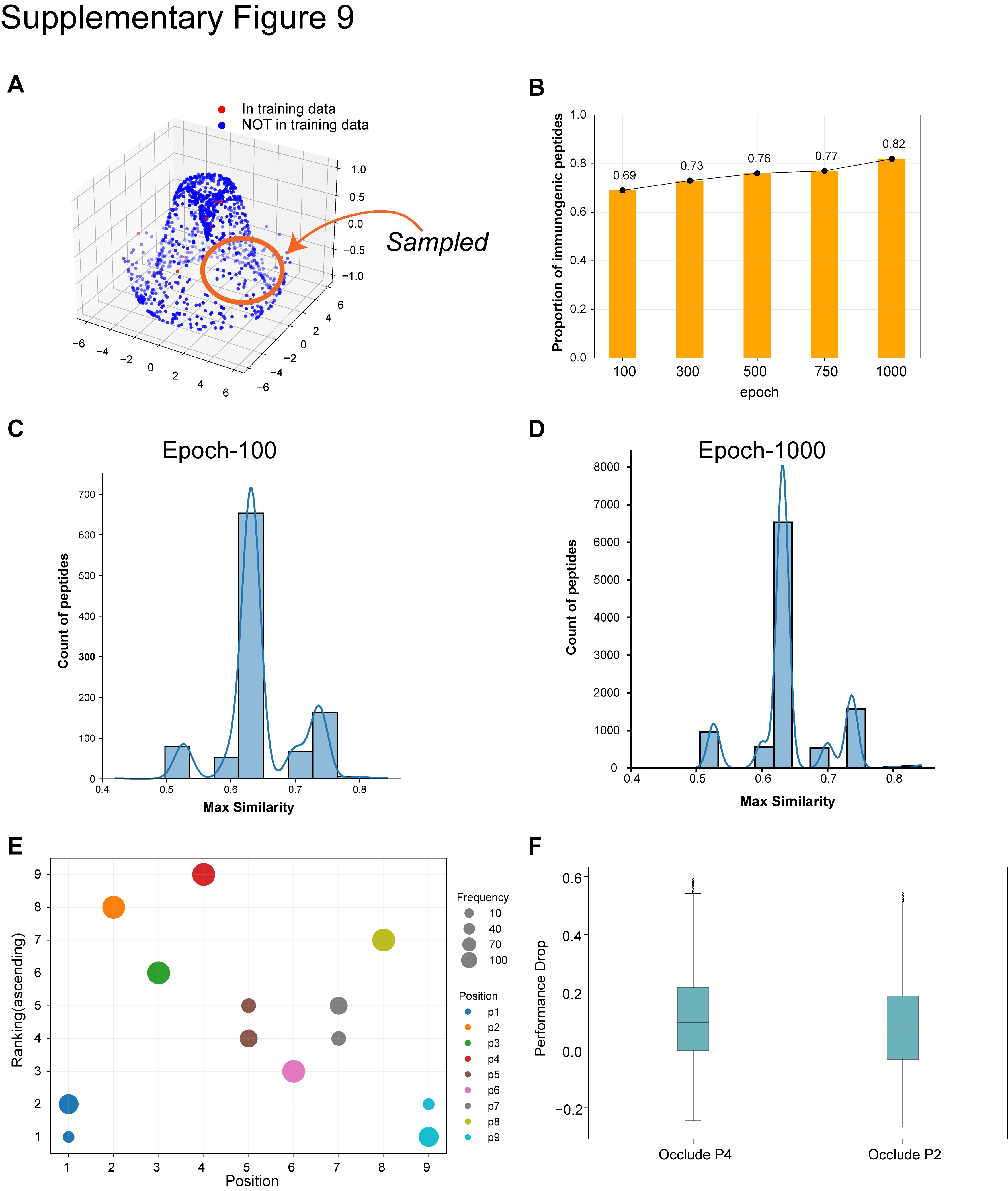

Supplement: FigureS9_bbab160 [file figures9_bbab160.jpeg]
