# Supplementary material for: DeepImmuno: deep learning-empowered prediction and generation of immunogenic peptides for T-cell immunity
Source: Brief Bioinform. 2021 May 3;22(6):bbab160. doi: 10.1093/bib/bbab160 (PMC8135853; doi:10.1093/bib/bbab160)
Supplement: Supplemental_Methods-r2_bbab160 [file supplemental_methods-r2_bbab160.pdf]

**DeepImmuno: Deep learning-empowered prediction and generation of immunogenic peptides for T cell immunity**

## SUPPLEMENTAL METHODS

Encoding Strategy

To represent each HLA allele and encoded peptide sequences in a numerical matrix as the input for each evaluated machine learning and deep learning algorithms, we developed and tested different encoding strategies. We used HLA paratopes (HLA-antigen interacting residues) as a proxy of different HLA alleles as these sequences contain the most salient information to describe peptide-HLA spatial interactions. The AAindex encoding strategy was designed to account for amino acid comprehensive physicochemical properties.

*AAIndex*: We retrieved 566 amino acid associated physicochemical properties from the AAindex1 database [15]. Among the 566 properties, 13 indices were discarded due to missing values for certain amino acids (**Supplementary Table 1**). We introduced a placeholder amino acid "-" for padding the gaps of HLA paratope sequences and 9-mer peptides (see below). The corresponding AAindex values were set as the average of all other 20 canonical amino acids. This method adds the total amino acid number to 21. The resulting 21 x 553 numeric matrix was normalized using RobustScaler [21] via the following operation:

$$X_{ij}^{new} = \frac{X_{ij}^{old} - m_j}{IQR_j}$$

Where **X** is the numeric matrix, **m** is the median per each feature column and **IQR** is the interquartile (Q3-Q1) per each feature column. The normalized feature matrix undergoes a principal component analysis (PCA) to remove noisy features such that it only retains relevant components. We chose 12 principal components which explain 95% total variance. This step leads to a 21 x 12 numerical matrix (hereafter AAindex matrix). For peptides, we adopted an encoding schema similar to that of O'Donnell et al [22] to pad shorter peptides (9-mer) to a longer sequence (10-mer) such that first five residues and the last four residues were joined by a placeholder "-", since the two termini are often involved in binding interactions [23,24]. For MHC molecules, we encoded each MHC allele based on its paratopes' sequence, which is the set of discontinuous residues sterically interacting with peptides. This paratope information was evidenced and analyzed from crystal structure and was retrieved from IMGT-3D-Structure database [25] (http://www.imgt.org/3Dstructure-DB/). For MHC alleles which did not have a solved peptide-MHC structure, their paratope information was determined by their neighbors. Specifically, the paratopes of allele HLA-A*2403 were determined by its nearest neighbor HLA-A*2402, which is a more frequent allele whose paratope sequence is available. We then performed two rounds of multiple sequence alignment using clustal-omega [26]. The first iteration was used for generating a consensus sequence for a single HLA allele from all its solved crystal structure, while the second round was for all paratope sequences with same length, gaps were filled with the placeholder "-" [26]. Example is shown in **Supplementary Figure 1**.

Beta Binomial immunogenic model

Rather than treat the classification of immunogenic peptides as a binary problem, we developed a beta-binomial approach to model the strength of the immunogenicity predictions based on the

variable experimental training evidence for specific HLA alleles and amino acid pairs. Rather than reflect the genuine biochemical immunogenicity strength, a benefit of such a model is that it can reflect the statistical confidence in our model based on the relative experimental evidence that exists in our training dataset. Hence, a beta binomial immunogenicity model will be able to account for the statistical confidence of each peptide in the training set (e.g., one peptide may have 40 validation points whereas another will have only 6).

Three columns of information from the IEDB database were used in the creation of the beta-binomial model, namely the immunogenic class (**x**), result claimed by submitter (positive, positive-high, positive-intermediate, positive-low, negative), number of subjects tested (**s**) and number of subjects responded (**s-f**). We derived a prior beta distribution based on the immunogenic class (**x**):

$$Prior = \begin{cases} Beta(3,3) & x = Negative \\ Beta(28,1) & x = Positive - low \\ Beta(30,1) & x = Positive - high \\ Beta(32,1) & x = Positive - Intermediate \quad OR \quad Positive \end{cases}$$

For a given epitope (data instance), assuming that we observe **s** successful T cell responses and **f** fails, then the posterior distribution of this epitope's immunogenic potential follow a new beta distribution:

$$Posterior = \begin{cases} Beta(3+S,3+F) & x = Negative \\ Beta(28+S,1+F) & x = Positive - low \\ Beta(30+S,1+F) & x = Positive - high \\ Beta(32+S,1+F) & x = Positive - Intermediate \quad OR \quad Positive \end{cases}$$

We then performed 50 bootstrapped iterations from the derived posterior distribution and used the average as the final immunogenic potential of a certain peptide-MHC complex.

Prediction models

We first adopted and rigorously compared the performance of five machine learning algorithms (ElasticNet, KNN, SVM, Random Forest and AdaBoost), after optimizing parameters for each method as follows. ElasticNet regression was first cross-validated to determine the best hyperparameters (alpha=0.01, l1_ratio=0.51), where alpha controlled the regularization strength and l1_ratio determined the percentage of the L1-norm penalty (lasso regression) and the L2-norm penalty (ridge regression). KNN regressor was cross-validated to determine the best hyperparameters (n_neighbors=23), n_neighbors control the neighbor information used for inferring query point's properties. SVM linear regressor was cross-validated to determine the best hyperparameter (C=0.01), C is the reciprocal of regularization strength which is inversely proportional to how many mistakes are allowed in the model. Random Forest was cross-validated to determine the best hyperparameter (n_estimators=200, min_sample_leaf=1), n_estimators control the number of decision trees in the model and min_sample_leaf control the minimum number of samples to be a leaf node. The aforementioned cross-validations were all 10-fold and rooted mean square error (RMSE) was used as default evaluation criteria if not specifically mentioned otherwise. The same hyperparameters were adopted for the adaptive boost (AdaBoost) model, similar to Random Forest, since they are both tree-based ensemble methods.

We further implemented and optimized three deep learning architectures:
*CNN*: The pictorial architecture is shown in **Figure 1C**. Peptide and MHC were processed by two consecutive convolutional layers, followed by two dense layers to consider the interactions between peptide and MHC. The basic convolution operation is mathematically represented as:

$$F_{d,j,k} = ReLu(\sum_{d}\sum_{i} X_{d+j,i}W_{d,i,k})$$

Where **F** is the resultant feature map, **X** is the input numerical matrix and **W** is the kernel. Lower case **d** denotes the row index and **i** denotes the column index of original matrix and **k** denotes the index of kernels in the convolutional layers. The **ReLu** function was used as an activation function. When training the model, we set the batch_size = 128. Two early stopping strategies were adopted: 1) monitor the training_loss with patience = 2; training will immediately stop if training loss increases and 2) monitor the validation_loss with patience=15; training will stop when we did not observe validation loss decrease in 15 epochs. To assess the potential effect of different training epochs, we disabled the early stopping and let the model stop at epoch 64, 100 or 150. Label shuffling was performed by randomizing all of the immunogenic and non-immunogenic peptide labels during the training phase. The detailed architecture of the CNN model is shown in Supplementary Table 4. We note that tuning with different filter sizes does not have obvious effects on model performance (data not shown).

*ResNet*: An overview is shown in **Supplement Figure 2A**. Peptide and MHC undergo three consecutive residue blocks, each residual block containing three CNN layers followed by a maxpool layer. Two dense layers were used at the end for prediction. Each residual block [27] contains skip connection which feed the input back to the output to avoid gradient vanishing as determined by:

$$Y_{i,j} = \alpha * Conv(X_{i,j}) + X_{i,j}$$

Where **Y** is the output matrix of a single residual block, **α** determines the fraction of convolutional output we want to keep, **X** is the original input matrix.

*GNN*: An overview is shown in **Supplementary Figure 2B**. Each peptide-MHC complex was represented by an acyclic undirected graph. Two types of edges were specified, ones were intra-edges denoting the interactions between/within-peptide and within-MHC interactions, others were inter-edges denoting the interactions between peptide and MHC. To emphasize the peptide-MHC interactions, we assigned a weight = 2 on inter-edges and weight = 1 on intra-edges. Two graph convolutional layers [28] were built upon the constructed graph objects, followed by a mean readout layer [29] to summarize node embedding at the graph level. The learned graph level features are fed into two dense layers for predictions. The core graph convolution operation can be mathematically described as:

$$\hat{A} = A_{ij} + I_N$$

$$D = \sum_j \hat{A}_{ij}$$

$$H^{l+1} = ReLu(D^{-\frac{1}{2}}\hat{A}D^{-\frac{1}{2}}H^lW^l)$$

Where $\mathbf{A_{ij}}$ is the adjacency matrix of graph objects, **i**-th row and **j**-th column represent the **i**th node and its **j**-th associated feature and $\mathbf{I_N}$ is the self-loop which is a diagonal matrix. The degree matrix **D** is the sum of adjacency matrix over the columns. **H** is the graph representation, which corresponds to a **N** x **M** matrix where **N** is the number of nodes and **M** is the number of features associated with each node. Lower case **i** denotes the layer of graph representation and **W** is the trainable weight matrix that governs the learning process.

SARS-CoV-2 Analysis

The SARS-CoV-2 proteome sequence of each open reading frame (ORF) was downloaded from NCBI SARS-CoV-2 portal (https://www.ncbi.nlm.nih.gov/sars-cov-2/). The ten most common HLA alleles in human were collected from a prior study [11], namely HLA-A*0101, HLA-A*0201, HLA-A*0301, HLA-A*1101, HLA-A*2402, HLA-B*0702, HLA-B*0801, HLA-B*1501, HLA-B*4001, HLA-C*0702. Each ORF was translated to protein sequences *in silico* and truncated to 9- and 10-mers, prior to DeepImmuno-CNN analysis. DeepImmuno-CNN predictions for all of the 10 most common HLA alleles were averaged to obtain a consensus score to assess a peptide's immunogenicity in the population.

SARS-CoV2-2 variant analysis was conducted by replacing amino acids of the spike protein at specific positions (D614G, E484K and N501Y). The 9-mer peptides that harbor the point mutation were supplied to the DeepImmuno-CNN web portal (https://deepimmuno.research.cchmc.org/) along with their normal counterpart to predict immunogenicity. The same mutation that resides at different positions of the peptide were queried separately.

Occlusion Sensitivity

To assess the relative importance of each amino-acid position in the model, we sequentially occluded those features associated with each position by setting the values = 0 and re-assessed performance by recording the decrease in resultant predictive score. We measured the performance decrease in all 4,143 positive training instances. We sampled 2,000 positive instances each time and measured the decrease in performance and a rank of position was derived and recorded in an array. Note that we did not retrain the initial model but rather zeroed-out/masked each position. We simulated this process 100 times to validate the robustness of the ranking information. A one-sided Mann Whitney U test was performed to test the statistical significance of each occlusion. The motif heatmap of specific MHC alleles were generated based on the schema proposed by Hu et al. [24], where a position-weighted matrix was produced from all collected immunogenic peptides of the queried MHC allele as described by:

$$H_{ij} = w_i * \sum_1^m \delta(a_j, j)$$

Where **H** is the resultant motif matrix, **w** is the position importance derived from occlusion analysis, **j** denotes 20 amino acids, **i** denotes the position index and **m** signifies the overall number of immunogenic peptides for the queried MHC allele and **δ** is an indicator function.

Benchmarking

We benchmarked DeepImmuno-CNN against two existing immunogenicity prediction tools with a high reported auROC. For deep learning-based methods, we benchmark against a Gated Recurrent Unit (GRU) [30] based deep learning model DeepHLApan [10]. We downloaded the docker file from docker hub (https://hub.docker.com/r/biopharm/deephlapan) specified in the github page and ran the software in a docker container. We also benchmarked against IEDB's default MHC-I immunogenicity prediction algorithm [8] from the IEDB web portal. Benchmarking results are shown in **Figure 2**. Other algorithms were excluded for evaluation due to either challenging to use interfaces (e.g. inability to query multiple alleles simultaneously - INeo-Epp [9]) or because they could not be directly compared due to underlying assumptions of the method (e.g., Neopepsee [7]). The evaluated algorithms were not time benchmarked, as the running time for all algorithms were relatively fast (seconds).

Generative Adversarial Network (GAN)

To determine whether immunogenic peptides could not only be predicted but learned and simulated, we trained a GAN model. The GAN model is composed of a generator and a discriminator. We adopted the architecture proposed by Gupta et al [13], as shown in **Figure 1D**. Briefly, an one-hot encoding strategy was used to facilitate the inverse transformation from a probability to pseudo-sequence, then five residual blocks were chained together in both the generator and the discriminator. A 1-dimensional convolutional layer was used to convert the number of channels to be the number of 21 amino acids sequences. We modified the general objective function using Wasserstein distance (WGAN) [17] and improved the stability of training by enforcing 1-Lipshitz constraint using a gradient penalty (WGAN-GP) [31]. The proposed GAN model uses the following loss function:

$$Loss = E_{x\sim P_g}[D(x)] - E_{x\sim P_r}[D(x)] + \lambda E_{x\sim P}[||\nabla x D(x)||_2 - 1]^2$$

Where $\mathbf{P_g}$ is the generated sequence, $\mathbf{P_r}$ is the real sequence, and **D(x)** indicates the predictive score from the discriminator.

We applied a previously described training strategy for the GAN [13]. Here, gumbel-softmax (tau=0.75) was used in lieu of ordinary softmax to allow sampling from the discrete output. Beta1 and Beta2 hyperparameters of the adaptive learning Adam optimization algorithm were set to 0.5 and 0.9 respectively. Finally, the parameters in the discriminator are updated every mini-batch, while the parameters in the generator are updated every 10 mini-batches. The model was trained using batch_size=64 and trained on 100 epochs.

Similarity between pseudo-sequence and real sequence

The similarity between two peptides' sequences was defined as the longest contiguous common sequence length between two queried sequences. For two sequence **S1** and **S2**, the similarity was computed as:

$$Similarity(S1, S2) = \frac{2 \times \sum M}{len(S1) + len(S2)}$$

Where **M** denotes the length of each longest common sequence (LCS). **S1** and **S2** belong to 20 amino acids plus a placeholder amino acid "-". We used the SequenceMatcher function in Python3 difflib package for calculation.

Web application development

We built an interactive web application (https://deepimmuno.research.cchmc.org/) for quick query of immunogenic epitopes. The front-end was implemented in HTML5 with bootstrap 4 framework. The back-end was implemented in the Flask python3 framework. The webpage was deployed to Heroku platform through the DeepImmuno GitHub web portal. The web logos are generated using (http://weblogo.threeplusone.com/create.cgi) for bound peptides of each MHC allele [32].

## REFERENCES


1. Nelde A, Bilich T, Heitmann JS, et al. SARS-CoV-2-derived peptides define heterologous and COVID-19-induced T cell recognition. Nat. Immunol. 2020;
2. Joglekar AV, Li G. T cell antigen discovery. Nature Methods 2020;
3. Tang S, Madhavan S. neoantigenR: An annotation based pipeline for tumor neoantigen identification from sequencing data. 2017; 171843
4. Tung C-W, Ho S-Y. POPI: predicting immunogenicity of MHC class I binding peptides by mining informative physicochemical properties. Bioinformatics 2007; 23:942–949
5. Tung C-W, Ziehm M, Kämper A, et al. POPISK: T-cell reactivity prediction using support vector machines and string kernels. BMC Bioinformatics 2011; 12:446
6. Saethang T, Hirose O, Kimkong I, et al. PAAQD: Predicting immunogenicity of MHC class I binding peptides using amino acid pairwise contact potentials and quantum topological molecular similarity descriptors. J. Immunol. Methods 2013; 387:293–302
7. Kim S, Kim HS, Kim E, et al. Neopepsee: accurate genome-level prediction of neoantigens by harnessing sequence and amino acid immunogenicity information. Ann. Oncol. 2018; 29:1030–1036
8. Calis JJA, Maybeno M, Greenbaum JA, et al. Properties of MHC class I presented peptides that enhance immunogenicity. PLoS Comput. Biol. 2013; 9:e1003266
9. Wang G, Wan H, Jian X, et al. INeo-Epp: A Novel T-Cell HLA Class-I Immunogenicity or Neoantigenic Epitope Prediction Method Based on Sequence-Related Amino Acid Features. Biomed Res. Int. 2020; 2020:5798356
10. Wu J, Wang W, Zhang J, et al. DeepHLApan: A Deep Learning Approach for Neoantigen Prediction Considering Both HLA-Peptide Binding and Immunogenicity. Front. Immunol. 2019; 10:2559
11. Kingma DP, Mohamed S, Jimenez Rezende D, et al. Semi-supervised learning with deep generative models. Adv. Neural Inf. Process. Syst. 2014; 27:3581–3589
12. Jin L, Tan F, Jiang S. Generative Adversarial Network Technologies and Applications in Computer Vision. Comput. Intell. Neurosci. 2020; 2020:1459107
13. Gupta A, Zou J. Feedback GAN for DNA optimizes protein functions. Nature Machine Intelligence 2019; 1:105–111
14. Gu J, Wang Z, Kuen J, et al. Recent advances in convolutional neural networks. Pattern Recognit. 2018; 77:354–377
15. Kawashima S, Pokarowski P, Pokarowska M, et al. AAindex: amino acid index database, progress report 2008. Nucleic Acids Research 2007; 36:D202–D205
16. Cerda P, Varoquaux G, Kégl B. Similarity encoding for learning with dirty categorical variables. Machine Learning 2018; 107:1477–1494
17. Martin Arjovsky SC, Bottou L. Wasserstein generative adversarial networks. Proceedings of the 34 th International Conference on Machine Learning, Sydney, Australia 2017;
18. Nielsen M, Lundegaard C, Blicher T, et al. NetMHCpan, a Method for Quantitative Predictions of Peptide Binding to Any HLA-A and -B Locus Protein of Known Sequence. PLoS ONE 2007; 2:e796
19. Weiskopf D, Angelo MA, de Azeredo EL, et al. Comprehensive analysis of dengue virus-specific responses supports an HLA-linked protective role for CD8+ T cells. Proc. Natl. Acad. Sci. U. S. A. 2013; 110:E2046–53
20. Wells DK, van Buuren MM, Dang KK, et al. Key Parameters of Tumor Epitope Immunogenicity Revealed Through a Consortium Approach Improve Neoantigen Prediction. Cell 2020; 183:818–834.e13
21. Nelli F. Machine Learning with scikit-learn. Python Data Analytics: With Pandas, NumPy, and Matplotlib 2018; 313–347
22. O'Donnell TJ, Rubinsteyn A, Laserson U. MHCflurry 2.0: Improved Pan-Allele Prediction of MHC Class I-Presented Peptides by Incorporating Antigen Processing. Cell Syst 2020; 11:418–419
23. Engelhard VH. Structure of Peptides Associated with Class I and Class II MHC Molecules. Annual Review of Immunology 1994; 12:181–207
24. Hu Y, Wang Z, Hu H, et al. ACME: pan-specific peptide-MHC class I binding prediction through

attention-based deep neural networks. Bioinformatics 2019; 35:4946–4954
25. Ehrenmann F, Kaas Q, Lefranc M-P. IMGT/3Dstructure-DB and IMGT/DomainGapAlign: a database and a tool for immunoglobulins or antibodies, T cell receptors, MHC, IgSF and MhcSF. Nucleic Acids Res. 2010; 38:D301–7
26. Sievers F, Wilm A, Dineen D, et al. Fast, scalable generation of high-quality protein multiple sequence alignments using Clustal Omega. Mol. Syst. Biol. 2011; 7:539
27. He K, Zhang X, Ren S, et al. Deep Residual Learning for Image Recognition. 2016 IEEE Conference on Computer Vision and Pattern Recognition (CVPR) 2016;
28. Kipf TN, Welling M. Semi-Supervised Classification with Graph Convolutional Networks. arXiv [cs.LG] 2016;
29. Monti F, Frasca F, Eynard D, et al. Fake News Detection on Social Media using Geometric Deep Learning. arXiv [cs.SI] 2019;
30. Cho K, van Merrienboer B, Gulcehre C, et al. Learning Phrase Representations using RNN Encoder-Decoder for Statistical Machine Translation. arXiv [cs.CL] 2014;
31. Gulrajani I, Ahmed F, Arjovsky M, et al. Improved Training of Wasserstein GANs. Advances in Neural Information Processing Systems 2017; 30:5767–5777
32. Crooks GE. WebLogo: A Sequence Logo Generator. Genome Research 2004; 14:1188–1190
33. Saito T, Rehmsmeier M. The Precision-Recall Plot Is More Informative than the ROC Plot When Evaluating Binary Classifiers on Imbalanced Datasets. PLOS ONE 2015; 10:e0118432
34. Geirhos R, Jacobsen J-H, Michaelis C, et al. Shortcut learning in deep neural networks. Nature Machine Intelligence 2020; 2:665–673
35. Chan KK, Dorosky D, Sharma P, et al. Engineering human ACE2 to optimize binding to the spike protein of SARS coronavirus 2. Science 2020; 369:1261–1265
36. Wucherpfennig KW, Call MJ, Deng L, et al. Structural alterations in peptide-MHC recognition by self-reactive T cell receptors. Curr. Opin. Immunol. 2009; 21:590–595
37. Rudolph MG, Stanfield RL, Wilson IA. How TCRs bind MHCs, peptides, and coreceptors. Annu. Rev. Immunol. 2006; 24:419–466
38. Wu D, Gallagher DT, Gowthaman R, et al. Structural basis for oligoclonal T cell recognition of a shared p53 cancer neoantigen. Nat. Commun. 2020; 11:2908
39. Kearns-Jonker M, Barteneva N, Mencel R, et al. Use of molecular modeling and site-directed mutagenesis to define the structural basis for the immune response to carbohydrate xenoantigens. BMC Immunol. 2007; 8:3
40. Morra G. Fast Python: NumPy and Cython. Lecture Notes in Earth System Sciences 2018; 35–60
41. Alipanahi B, Delong A, Weirauch MT, et al. Predicting the sequence specificities of DNA- and RNA-binding proteins by deep learning. Nature Biotechnology 2015; 33:831–838
42. Pan X, Shen H-B. Predicting RNA-protein binding sites and motifs through combining local and global deep convolutional neural networks. Bioinformatics 2018; 34:3427–3436
43. Gowthaman R, Pierce BG. TCR3d: The T cell receptor structural repertoire database. Bioinformatics 2019; 35:5323–5325
44. Bagaev DV, Vroomans RMA, Samir J, et al. VDJdb in 2019: database extension, new analysis infrastructure and a T-cell receptor motif compendium. Nucleic Acids Res. 2020; 48:D1057–D1062
45. Azizi E, Carr AJ, Plitas G, et al. Single-Cell Map of Diverse Immune Phenotypes in the Breast Tumor Microenvironment. Cell 2018; 174:1293–1308.e36
46. Vieyra-Lobato MR, Vela-Ojeda J, Montiel-Cervantes L, et al. Description of CD8 Regulatory T Lymphocytes and Their Specific Intervention in Graft-versus-Host and Infectious Diseases, Autoimmunity, and Cancer. J Immunol Res 2018; 2018:3758713
47. De Boer RJ, Perelson AS. T cell repertoires and competitive exclusion. J. Theor. Biol. 1994; 169:375–390
